# Prenatal programming of environmental sensitivity

**DOI:** 10.1038/s41398-023-02461-y

**Published:** 2023-05-10

**Authors:** Sarah Hartman, Jay Belsky, Michael Pluess

**Affiliations:** 1 Department of Human Eology, University of California, Davis, CA USA; 2 Department of Psychological Sciences, School of Psychology, University of Surrey, Guildford, UK; 3 Department of Biological and Experimental Psychology, School of Biological and Behavioural Sciences, Queen Mary University of London, London, UK

**Keywords:** Human behaviour, Molecular neuroscience

## Abstract

According to several theories, people differ in their sensitivity to environmental influences with some more susceptible than others to both supportive *and* adverse contextual conditions. Such differences in *environmental sensitivity* have a genetic basis but are also shaped by environmental factors. Herein we narratively build on our previous work proposing that prenatal experiences contribute to the development of environmental sensitivity. This hypothesis of *prenatal programming of postnatal plasticity* has considerable empirical support. After presenting illustrative animal and human evidence consistent with this claim, we discuss a range of biological mechanisms likely involved in the pathway from prenatal stress exposure to postnatal environmental sensitivity. We also consider work suggesting that genetic differences, gender, as well as the timing, duration and intensity of prenatal exposures may moderate the effects of prenatal programming on postnatal environmental susceptibility or sensitivity. Before concluding, we highlight “unknowns in the prenatal programming of environmental sensitivity” and their practical implications. Ultimately, we conclude that prenatal stress does not necessarily predispose individuals to problematical development, but rather increases sensitivity to both adverse and supportive postnatal contexts. Thus, prenatal stress may actually foster positive development if paired with supportive and caring postnatal environments.

## Introduction

It can be widely observed that people differ in how they respond to experiences, both in the short and longer term; the emphasis herein is on the latter. Consider, for example, that while some seem generally unperturbed by their experiences and exposures, others appear deeply affected. One reason for such variation is that individuals differ in their general susceptibility or sensitivity to environmental influences, such that some prove more and others less sensitive or susceptible. Several theories advanced over recent decades independently made a case for the existence of such individual differences [1–3] and have been summarized in the integrative framework of *environmental sensitivity* [4]. Importantly, according to these theories, heightened sensitivity is not only associated with increased vulnerability to the negative effects of adversity, but also with greater propensity to benefit from supportive experiences [5]. Although differences in general sensitivity are understood to have a genetic basis [6], environmental factors play an important role, too, including as early as during pregnancy [7, 8], the focus of this paper. In what follows, we present theory and empirical evidence consistent with this assertion and thus *prenatal programming of environmental sensitivity*. After considering theories and mechanisms of susceptibility to environmental influences, we discuss theoretical perspectives regarding the role of prenatal factors implicated in the development of such sensitivity. This sets the stage for the presentation of illustrative empirical evidence for prenatal stress affecting environmental sensitivity, including discussion of potential biological mechanisms and moderating factors, all of which stimulates practical implications and directions for future research.

## Theories of environmental sensitivity

The notion that people differ in their sensitivity to environmental influences is not new in the field of psychiatry. Indeed, the *diathesis-stress* model is well established [9, 10], stipulating that some people are more *vulnerable* to the *negative* effects of *adverse* or stressful experiences due to inherent personal characteristics (e.g., genetics, physiology, temperament). Notably, over recent decades new perspectives have emerged indicating that some of these personal attributes are associated with sensitivity to both negative *and* positive experiences [11, 12]. In other words, some people are not only more vulnerable when faced with stressful experiences, but also benefit disproportionately from positive ones, such as supportive parenting [13] and psychological intervention [14], as the function of a general heightened sensitivity to environmental quality.

Such differences in environmental sensitivity have been central to the theory of *differential susceptibility* [1, 15], according to which natural selection has produced variation in susceptibility to environmental influences: Whereas some children are generally more unaffected—over the longer term—to would-be environmental influences during development (i.e., they are relatively “fixed”), others are more sensitive to the quality of their context and therefore more impacted in their development by both negative *and* positive exposures (i.e., they are relatively more “plastic”). Notably, the framework of *Biological Sensitivity to Context* [2] describes similar variation in environmental sensitivity, but, distinctively, regards such variation as resulting from the early rearing environment: those growing up in particularly adverse or especially supportive contexts develop enhanced sensitivity resulting from heightened physiological reactivity compared to those raised in more intermediate environments. Whilst the former two theories are strongly based on developmental and evolutionary considerations [16], a third theory of *Sensory Processing Sensitivity* [3, 17] is based on a psychological approach without detailed consideration of actual origins, stipulating that a minority of the population is characterized by a stable personality trait reflecting heightened sensory sensitivity and deeper cognitive processing of internal and external stimuli.

What these three theories have in common, in contrast to traditional *diathesis-stress* thinking, is that they contend that individuals do not only differ in their response to negative but also in response to positive exposures. A fourth theory is the converse of the diathesis-stress model in that *vantage sensitivity* stipulates that some individuals benefit more and others less from supportive environments due to differences in their sensitivity [5]. Given that these theories describe differences in susceptibility to environmental influences, they have been combined into a broader integrative framework of *Environmental Sensitivity* (which also includes vantage sensitivity) [4]. For simplicity, we will refer to environmental sensitivity or susceptibility from here on rather than to the individual theories.

## Mechanisms of environmental sensitivity

Multiple empirical studies [for review, see [1, 18, 19]] documenting environmental sensitivity have linked heightened sensitivity with diverse individual characteristics, including genetic [20, 21], physiological [22, 23], and behavioural-psychological factors [12, 24, 25]. The different theories of environmental sensitivity all agree that susceptibility is most likely driven by features of the central nervous system [3, 4, 16, 26], including structural [27, 28] and functional aspects of the brain [29–31], often the result of physiological reactivity, sensory sensitivity and cognitive processing of contextual information. According to this *neurosensitivity* hypothesis [32], an individual’s sensitivity of the central nervous system is shaped by the complex interplay of genetic and environmental factors and becomes manifest in physiological reactivity and behavioural traits (i.e., infant temperament). Indeed, findings from a large twin study indicates that 47% of differences in environmental sensitivity, measured in adolescents with the *Highly Sensitive Child* (HSC) scale [25], are explained by genetic factors and the remaining 53% by environmental influences [33]. Although not much is known yet regarding the specific environmental factors that contribute to the development of susceptibility to environmental influences, there is evidence that environmental influences during the prenatal period, such as maternal stress or exposure to traumatic experiences, may play an important role in shaping physiological and behavioural factors associated with environmental sensitivity [7, 34, 35]. The basic notion that prenatal experiences can shape stable phenotypic characteristics of an individual has been the focus of much work conducted under the terms of *developmental origins of health and disease* [36, 37] as well as *prenatal* or *fetal programming* [38, 39].

## Prenatal programming

According to the dominant view of prenatal programming, nutritional and hormonal cues reflective of the quality of the mother’s current and perhaps even childhood environment are passed on to the fetus in utero through the placenta. In so doing, they shape (i.e., “programme”) the fetus’ metabolism and stress reactivity, amongst other processes, in preparation for the conditions of the postnatal environment [40, 41]. The basic understanding is that this will (probabilistically) promote optimal adjustment by “fitting” the fetus to the postnatal environment it is most likely to encounter. When, however, there is a “mismatch” between prenatally “programmed” features of the child and the quality of the postnatal environment, such programming effects are expected to adversely affect the child’s development [40, 41]. This is likely to happen when the actual postnatal environment differs substantially from the environment the mother experienced during pregnancy or earlier. Thus, what was once evolutionarily adaptive under the assumption of prenatal-postnatal environmental stability is no longer so when the prenatal environment is not predictive of postnatal conditions. Similarly, more recent theories including Predictive Adaptive Response (PAR) [42] expand on this thinking to address the adaptive reasons of why organisms would adjust their development dependent on cues received in the prenatal environment.

A different explanation for such adverse prenatal programming effects is that the environmental “cues” that the fetus receives in utero affords “insight” into the *degree of stability* of the postnatal context [7, 8]. Should the environment be relatively unstable and more likely to change, it would make sense for the fetus to develop a higher degree of general environmental sensitivity in order to be better able to adapt to a wider range of postnatal environments [43]. Thus, with this line of thinking, prenatal stress may adversely affect offspring development under conditions of postnatal adversity but also foster more positive development when a supportive postnatal environment is encountered. In accordance with this hypothesis, several physiological and behavioural traits that have been associated with heightened environmental sensitivity (e.g., cortisol stress reactivity, difficult temperament) have also been shown to be shaped, at least partially, by prenatal factors such as maternal stress and other exposures during pregnancy [for review of empirical evidence, see [7, 8]].

In what follows, we review the strongest and most recent empirical evidence for the hypothesis that prenatal factors shape individual differences in environmental sensitivity, drawing on both animal and human studies. We then discuss several biological mechanisms that are likely involved in the prenatal programming of environmental sensitivity before considering whether such prenatal-programming effects are influenced by the intensity or timing of prenatal-stress exposure as well as child sex and genetic factors. It is important to note that this review is not intended to be a systematic examination of the field and although we present illustrative evidence for the proposed hypothesis, not all published studies are consistent with this thinking. Because of space constraints and the extensive literature that yields results consistent with environmental sensitivity, we decided this was the best way to construct this report. Thus, this paper builds upon previous research that provides empirical support for the aforementioned theoretical frameworks of sensitivity (e.g., differential susceptibility, diathesis-stress, vantage sensitivity) as well as for negative emotional temperament and increased stress reactivity as indicators of increased environmental sensitivity.

## Empirical evidence of prenatal programming of environmental sensitivity

Prenatal stress has been associated with detrimental outcomes, especially increased risk for psychopathology [44]. Although a majority of studies control for potential postnatal confounds (e.g., postpartum maternal depression, parenting quality [45]), fewer examine effects of both the prenatal and postnatal environment which is needed when investigating prenatal effects on environmental sensitivity. We turn next to both animal models and humans in order to highlight associations between prenatal stress and individual differences in sensitivity to postnatal exposures.

## Animal studies

Considering the numerous confounders present in human studies (e.g., comorbidities, strong pre/postnatal continuity) to say nothing of how unethical it would be to induce prenatal stress in humans, animal models are crucial to establish causal relationships between prenatal stress and postnatal functioning. Furthermore, animal models afford control over both the prenatal and postnatal environments which is critical considering that, in humans, prenatal and postnatal stress tend to co-occur [46]. In particular, cross-fostering experiments in which offspring are removed at birth from their biological parents and are reared by unrelated lactating dams or breeding pairs are a highly effective method for separating prenatal and genetic influence from postnatal conditions.

Prenatal stress has been shown to increase sensitivity, particularly vulnerability, to negative postnatal environments. Consider, for example, work using two different mouse strains, C57BL/6J and BALB/cJ, along with a pre- and postnatal cross-fostering design, to illuminate effects of prenatal and postnatal environments on adult anxiety-like behaviours [47]. C57BL/6J mice are known to have high levels of sociability, low levels of anxiety, and evince greater maternal care, whereas BALB/cJ mice are characterized by the opposite profile [47, 48]. In one study using prenatal cross-fostering, C57BL/6J embryos were transferred either to C57BL/6J (i.e., low anxiety) or BALB/cJ (i.e., high anxiety) females and, for postnatal cross-fostering, pups were moved after birth to either C57BL/6J (i.e., high care) or BALB/cJ (i.e., low care) dams. Although the investigators did not test behaviour or physiology during pregnancy, being cross fostered in utero to BALB/cJ mice can be considered a high prenatal stress condition given the strain’s characteristically high levels of anxiety-like behaviour compared to C57BL/6J. Results revealed that pups prenatally cross fostered to BALB/cJ females (i.e., prenatal stress) were more sensitive to postnatal rearing conditions. Specifically, prenatally stressed mice cross-fostered to BALB/cJ (i.e., low care) rearing mothers displayed the highest anxiety-like behaviour while pups not prenatally stressed were not affected by postnatal rearing condition.

Similarly, Hougaard et al. [49] found that rats prenatally stressed by either chronic mild stress (i.e., a variable schedule of mild stressors like damp bedding or crowding) or dexamethasone (i.e., a pharmacological stressor) that were subjected postnatally to a “stressful event” (i.e., blood sampling) later displayed greater reactivity (i.e., startle response) compared to rats not prenatally stressed. This is consistent with other research showing prenatally stressed rats, compared to controls, display heightened reactivity to contextual cues after fear conditioning [50].

Regarding individual differences in response to positive environmental influences [i.e., vantage sensitivity, [5]], Smythe and colleagues [51] examined the interaction between prenatal stress, induced by restraint, and postnatal handling on hypothalamic–pituitary–adrenal axis (HPA) axis activity in rats. Postnatal handling for brief periods is considered to be a positive early life experience as maternal care increases during the reunion with the mother after the handling [52]. Study results showed significant interactions between prenatal and postnatal conditions such that prenatally stressed rats assigned to postnatal handling had significantly lower corticotrophin-releasing hormone (CRH) levels in the Median Eminence whereas handling had no effect on rats that were not prenatally stressed. Moreover, other work produced similar evidence that rats exposed to prenatal stress, induced by unpredictable noise and light, are more sensitive to postnatal handling conditions with regard to adult anxiety-like behaviour than control rats who proved to be unaffected by postnatal handling [53].

Especially notable is recent work showing that prenatal stress increases offspring susceptibility to both negative and positive postnatal exposures, in that experimentally induced prenatal stress increased prairie voles postnatal sensitivity to low or high parenting quality [54]. Design-wise, pregnant voles were exposed to either a social stressor or not and, shortly after birth, pups were cross fostered to either high-quality (i.e., positive environment) or low-quality (i.e., negative environment) rearing conditions. Exposure to prenatal stress increased sensitivity to parenting quality in that prenatally stressed voles cross-fostered to high-quality parenting displayed the least behavioural and physiological reactivity as adults and the most reactivity if cross-fostered to low-quality parenting. Once again there was no effect of postnatal rearing on voles not prenatally stressed, thereby indicating lower susceptibility to the postnatal conditions. Clearly, then, animal work repeatedly indicates that prenatal stress can enhance environmental sensitivity to both positive and negative experiences. However, it is important to note that although research is still limited, epigenetics—which is discussed in the later section on “Potential Mechanisms” —is likely to play an important mechanistic role in these relations.

## Human studies

When Pluess and Belsky [7] first postulated their prenatal-programming-of-postnatal-plasticity hypothesis, they provided empirical evidence from the NICHD Study of Early Child Care that linked prenatal stress—indexed via low birth weight—to infant negative emotionality which, in turn, was associated with infants being more susceptible to effects of both low and high parenting quality on cognition and behaviour [7]. Relatedly, work by Sharp and associates [55] revealed that maternal prenatal anxiety, measured during late pregnancy, increased children’s developmental responsiveness to postnatal maternal stroking during the first few weeks of life on later anxious/depressive symptoms. In this case--children—and especially girls—exposed to high levels of prenatal maternal anxiety evinced greater anxious/depressive symptoms when they experienced limited maternal stroking postnatally, yet very little symptomology when exposed to a great deal of maternal stroking. The same was not true of children whose mothers experienced little anxiety during pregnancy; that is, they proved relatively immune to the effects of stroking.

Other research yielded similar results when exploring the interactive effects of prenatal stress and early caregiving on child functioning. For example, Grant et al. [56] found that the impact of maternal sensitivity on infant physiological reactivity during a stressful experience (i.e., still-face procedure) was greater for prenatally stressed infants (i.e., mothers with prenatal anxiety disorders) compared to less prenatally stressed infants. Specifically, prenatally stressed infants displayed the least reactivity to a stressful experience when maternal sensitivity was high but the most if maternal sensitivity was low, with response to maternal sensitivity diminished for infants not exposed to prenatal stress.

Especially notably is evidence from a natural experiment. Prenatal stress effects resulting from the Queensland flood in 2011 interacted with maternal emotional availability during infancy in predicting toddlers’ language development [57]: children of highly emotionally available mothers exposed to disaster-related stress during pregnancy and PTSD symptoms had better language development but, if mothers were low in emotional availability and been exposed to comparable stress their offspring had poorer language development. Yet again, parenting quality proved unrelated to toddler’s development for children exposed to low prenatal stress.

Additional research on prenatal programming of environmental sensitivity compares infants born pre-term versus full-term, low versus normal birth weight, and small-for versus normal gestational age. This substantial body of work shows that psychosocial stress is an aetiological risk factor for preterm birth, low birth weight, and being small-for-gestational age [58, 59]—even when controlling for other well-known risk factors [60, 61]. Thus, preterm birth, low birth weight, and being small-for-gestational age are considered markers of prenatal stress.

Pertinent to the issue of prenatal programming, then, is work which examined differential effects of postnatal caregiving on pre-term and full-term infants’ cognitive and social functioning [62]. Results revealed that preterm infants were more developmentally responsive to their caregiving environment, evincing the best and poorest social and cognitive functioning when exposed, respectively, to high- and low-quality caregiving, with full-term infants proving insensitive to these caregiving effects. Clearly, these findings are in line with those of earlier work which chronicled stronger associations between maternal responsiveness and cognitive growth in the case of preterm infants compared to full-term ones [63]. In fact, an intervention designed to promote maternal responsiveness proved successful in doing so but, consistent with the data summarized through this point. Notably, though, when it came to effects on children’s development, the benefits of being in the experimental group rather than the control group proved greater in the case of children born preterm [64].

Another relevant investigation found, intriguingly, that the effects of maternal sensitivity during childhood on adult wealth were moderated by whether children were born small or appropriate for gestational age [65]. Specifically, children small for gestational age—reflecting greater prenatal stress—proved more susceptible, in terms of their adult wealth, to the quality of maternal care during childhood than those who were appropriate for gestational age. Critically, only for those small for gestational age, did greater childhood maternal sensitivity predicted greater adult wealth whereas less sensitive parenting predicted lower adult wealth. Finally, in another investigation, low birth-weight children proved to be especially sensitive to low, but not high, postnatal maternal sensitivity [66]: low birth-weight children had poorer academic achievement if exposed to low levels of maternal sensitivity but did not differ in academic achievement from normal birth weight children when maternal sensitivity was high.

## Potential mechanisms

**Fig. 1:**
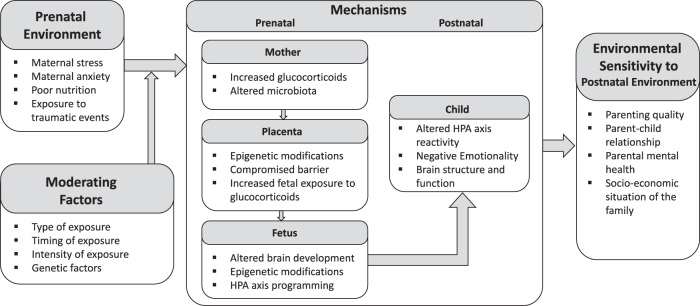
Graphic illustration of the pathways from prenatal stress to environmental sensitivity in the postnatal period. Effects of the prenatal environment are moderated by various characteristics of the exposure and the child, and programme an individual’s postnatal environmental sensitivity through several potential biological mechanisms.

We now consider several biological systems by which maternal distress may alter fetal development potentially instantiating environmental sensitivity. Given the ubiquitous effects of prenatal stress and thus numerous possible mechanisms, the focus here is admittedly limited; a more detailed review can be found elsewhere [8]. Although the candidate mechanisms are discussed separately, they are likely all related, as indicated in Fig. 1.

### Hypothalamic–pituitary–adrenal (HPA) axis

Two separate lines of research have highlighted the HPA axis as a key mediator of (1) environmental sensitivity and (2) prenatal stress effects on offspring. First, Boyce and Ellis [2] proposed heightened reactivity of the HPA system as the key mechanism for enhanced sensitivity to environmental effects. Indeed, heightened stress reactivity in children has been tied to greater sensitivity to both positive and negative experiences [8]. Second, prenatal programming of the offspring’s HPA axis functioning has been extensively investigated as a central mediator of maternal distress on offspring development in both human and animal models. For instance, a recent meta-analysis indicated that prenatal stress was associated with increased offspring glucocorticoids across 14 vertebrate species. Thus, not only is HPA axis sensitivity to prenatal stress highly conserved across evolution but, in addition, animal models of prenatal-HPA axis programming are appropriate guides for studying similar effects in humans [67].

In humans, various types of prenatal stress exposure are associated with altered child HPA axis functioning and stress reactivity [8]. For example, two meta-analyses found that dysregulation in child cortisol levels were predicted by (a) greater maternal cortisol during pregnancy [68] and (b) a variety of stressors experienced prenatally, including substance abuse and maternal distress [69]. This is consistent with animal studies finding that prenatal stress results in elevated levels of basal and reactive corticosteroids and decreased negative feedback of the HPA axis [70, 71]. Furthermore, in mice, prenatal stress affects stress neurocircuitry by increasing corticotropin-releasing factor in the amygdala and reducing hippocampal glucocorticoid receptor expression [72]. Similarly, in humans, prenatal stress is known to affect brain areas such as the prefrontal cortex, hippocampus, and amygdala that also regulate the HPA axis [73]. Although most of the reviewed evidence points to a pattern of heightened stress reactivity in response to prenatal stress, it is important to note that some work documents a more general dysregulation (i.e., blunting or over reactivity) in response to prenatal stress (e.g., [74]).

Given (a) the link between prenatal stress and stress reactivity and (b) that stress reactivity has been highlighted as an indicator of greater environmental sensitivity, it stands to reason that prenatal stress would foster greater physiological reactivity and, thereby, increased susceptibility to environmental influences (i.e., prenatal stress → greater physiological reactivity → increased sensitivity). Although portions of this process have been studied in isolation, the entirety of this potential mechanistic pathway has yet to be evaluated empirically.

### Negative emotionality

Akin to physiological reactivity, there is considerable evidence that negative emotionality in infancy reflects increased environmental sensitivity such that more negatively emotional infants are more sensitive to their rearing experiences [7]. Like physiological reactivity, then, negative emotionality has been consistently associated with prenatal stress. Notably, there is extensive evidence that such negative emotionality is itself a consequence of prenatal stress. Consider, for example, another natural experiment, this one of pregnant women exposed to the 1998 Canadian ice storm and who experienced heightened subjective distress or illness/infection at various time points in their pregnancy. They had infants with more difficult temperaments than pregnant women also exposed to the storm but who experienced less subjective distress or illness/infection [75]. Then there is a recent meta-analysis showing that prenatal stress, indexed by maternal psychological distress or exposure to major life events and natural disasters, is associated with greater child negative affectivity [76].

### Brain structure and function

Numerous studies have documented the effects of prenatal stress on both the structure and function of the brain [77]. Prenatal stress is associated with structural changes in the frontal and temporal lobes, including cortical thinning [78] and reductions in grey matter volume [34], as well as alterations in the limbic system, including increases in amygdala volume [79] and decreased hippocampal volume [80]. Functional brain activity is also affected by prenatal stress, with research chronicling reduced connectivity between the amygdala and prefrontal cortex in preterm infants exposed to prenatal maternal depression [81].

Importantly, differences in brain structure and function are thought to play a critical role in modulating environmental sensitivity [3, 31]. Evidence consistent with this claim comes from work showing that amygdala volume moderates the association between general environmental quality and child behaviour in boys [23]. Specifically, boys with larger amygdala volumes were more susceptible to beneficial effects of positive environmental features vis-à-vis their behaviour than boys with smaller amygdala volumes. Likewise, neonatal brain volume also moderates the association between parenting quality and cognitive development: newborns with larger brain volumes are more susceptible to effects of both positive and negative parenting on cognitive functioning [27].

### Epigenetic modifications

Epigenetics is considered a central molecular pathway by which early life stress is biologically embedded, contributing to individual differences in environmental sensitivity [82]. The term epigenetics refers to a process that produces changes in gene activity (activation or suppression) without alterations to the DNA. Epigenetic mechanisms include many processes but the most widely studied is DNA methylation (which suppresses gene expression). Most epigenetic studies have focused on the programming effects of early *postnatal* life showing, for example, that early postnatal stress in rats influences offspring hippocampal DNA methylation in the promoter region of *NR3C1*, the gene coding the glucocorticoid receptor (GR), which, among many other factors, regulates the stress response [83]. Similarly, in humans, emerging evidence indicates that quality of maternal care is related to offspring methylation in *N3CR1* as well as genes related to neuronal development (*BDNF)*, the serotonergic system (*SLC6A4*), and social behaviour (*OXTR*) [84].

Notably, research in both humans and animals suggests that prenatal stress may induce the same epigenetic modifications in homologous promoter regions of *NR3C1*. For example, prenatal stress increases offspring stress reactivity and hypothalamic methylation in the promoter region of *NR3C1* in mice [72]. Several human studies using neonatal cord blood find that prenatal anxiety [85], maternal exposure to interpartner violence [86], and depressive symptoms [87, 88] are associated with differential methylation patterns in the promoter region of *NR3C1*. In fact, one recent investigation examining pregnant mothers exposed to chronic stress found that placentas and neonatal cord blood had differential methylation patterns across several genes (i.e., *CRH*, *CRHBP*, *NR3C1*, and *FKBP5)* shown to regulate the HPA axis [89]. Notably, these methylation patterns were associated with lower infant birth weight, a marker of prenatal stress.

### Placental functioning

The placenta plays a central role as a modulator of maternal-fetal physiology and is, thus, a key candidate for mediating effects of maternal distress on the fetus. Specifically, the placenta reduces fetal exposure to maternal glucocorticoids through the placental barrier enzyme 11β-hydroxysteroid dehydrogenase Type II (11β-HSD2) which converts glucocorticoids into inactive metabolites such as cortisone. In rats, prenatal stress, induced by chronic restraint, is associated with a reduction in the expression and activity of the placental *11β-HSD2* [90]. Consequently, these epigenetic changes in placental *11β-HSD2* are themselves related to *11β-HSD2* methylation in the fetal brain [90].

In humans, greater maternal anxiety measured one day prior to birth predicts lower gene expression of placental *11β-HSD2* [91]. In another study, self-reported maternal stress during mid-gestation was associated with increased placental DNA methylation of 11β-HSD2 which, in turn, was associated with a reduction in fetal coupling, a marker of neurobehavioral development [92]. Other work has also tied decreased activity of placental 11β-HSD2 with altered fetal development, including fetal growth restriction [93], prematurity [94] and low birth weight [95].

Considered together, it appears that prenatal stress may diminish the placental barrier via epigenetic changes in *11β-HSD2*, thereby resulting in increased fetal exposure to maternal cortisol, with consequences for phenotypic outcomes.

### Intestinal microbiota

There is ever more evidence that intestinal microbiota influence brain development and behaviour via the microbiome-gut-brain axis. For example, alterations in the microbiome have been linked to psychological disorders including depression and anxiety [96]. Other work has highlighted variations in the composition of the intestinal microbiome as a potential marker of environmental sensitivity [97].

Evidence also indicates that establishment and development of the intestinal microbiota begins early in life. Birth can significantly influence the microbiome with infants born via vaginal delivery showing a bacterial composition resembling their mothers’ vaginal microbiome; babies born via Caesarean section show more skin-like microbiomes [98]. Moreover, emerging rodent work indicates that microbiome transmission can happen prenatally, possibly through the placental barrier or fetal ingestion of amniotic fluid [99].

Findings from several studies indicate that prenatal stress may influence such early maternal-infant transmission. Prenatal stress is known to alter the composition of the maternal microbiota [100] and is associated with differences in infant intestinal microbiota [101, 102]. For example, preterm birth status, a marker of prenatal stress, is associated with alterations in the infant microbiome [101, 103]. Additionally, maternal prenatal anxiety is associated with differences in microbiota found in newborn meconium [104]. Furthermore, both subjective reports of stress and cortisol exposure during pregnancy predicted differences in infant microbiota diversity which, in turn, was linked to infant health [105].

## Potential moderators

Having highlighted some mechanisms which may link prenatal stress and increased susceptibility to environmental influences, we now turn to potential moderators that may enhance or reduce the effect of prenatal exposures on environmental sensitivity. In other words, there is no claim that the link between prenatal stress and environmental sensitivity is inevitable. Like so much of development, “it depends”.

### Genetic moderation

Ample evidence indicates that prenatal stress may affect offspring differently depending on their genetic make-up [106]. Candidate genes have been evaluated for their possible roles in this interaction with the gene encoding serotonin transporter (*5-HTTLPR*) being one of the most heavily studied. For example, Pluess et al. [35] found that prenatal anxiety predicted greater negatively emotional temperaments but only for children carrying the *5-HTTLPR* short allele. Similarly, Babineau and colleagues [107] extended this work upon examining the interaction of prenatal depression and *5-HTTLPR* in predicting infant and early childhood behavioural dysregulation. Results showed that greater prenatal depression measured mid- or late pregnancy predicted more infant and early childhood dysregulation from 3–36 months of age, but only for short-allele *5-HTTLPR* carriers.

Other work has moved beyond examining variations in single candidate genes to a more systemic approach using polygenic scores based on multiple genetic variants [108, 109]. Belsky and Beaver [110] were the first to adopt this approach, discovering that the more would-be plasticity alleles—based on prior candidate gene work—moderated the effect of parenting on the self-control of teenage boys, such that the more plasticity alleles the adolescent carried, the more susceptible he appeared to parenting (in a manner consistent with differential susceptibility theorizing). For example, Silveira et al. [109] evaluated whether a polygenic score, based on genes co-expressed with the serotonin transporter gene (*SLC6A4*) in the hippocampus, moderated the relation between prenatal adversity and neurodevelopmental outcomes. Findings showed that exposure to greater prenatal adversity predicted a number of neurodevelopmental outcomes but only for children scoring high on the polygenic score. Additionally, Green et al. [111] observed that prenatal depression measured mid- to late pregnancy interacted with a polygenic profile score based on variants located in the *5-HTTLPR* and the dopamine-receptor D4 (*DRD4*) genes in predicting infant negative temperament. Results indicated that prenatal depression only predicted greater infant negative emotionality for those with higher polygenic sensitivity. Moreover, Qiu et al. [112] reported that a polygenic risk score for major depressive disorder moderated the association between prenatal maternal symptoms of depression and children’s hippocampal and amygdala volume. Specifically, higher polygenic risk predicted greater right hippocampal and amygdala volumes when children were exposed to high levels of prenatal depression.

### Sex

Although some research indicates that sex is a significant moderating factor of prenatal stress effects, findings are mixed with regard to which sex proves more susceptible. One inquiry, using data from pregnant mothers who were exposed to the 2011 Queensland Flood, revealed that higher levels of hardship during pregnancy predicted greater infant irritability, but only for boys [113]. Additional work reveals that boys have an increased risk of developing childhood attention-deficit/hyperactivity disorder (ADHD) if their mother lost a spouse or child during pregnancy [114]. Other studies show girls as evincing more compromised mental health outcomes [55], perhaps especially in the case of evidence documenting a significant association between maternal prenatal depression and risk for adult depression in women but not men [115].

These mixed findings may partly be due to sex-dependent differences in the outcome of interest. For example, boys present more frequently with intellectual impairment and childhood behavioural disorders related to prenatal stress whereas girls may develop subtler, later-onset anxiety and affective disorders in response to the same exposure [116].

### Type, timing, and intensity of prenatal exposure

There is an abundance of evidence that many different types of prenatal stress can influence child development, including maternal anxiety and depression [117, 118], pregnancy-specific anxiety [119] and exposure to acute disasters such as ice storms [120] or the 9/11 terrorist attacks [74]. This diversity of stressors also extends to animal work, some of which include repeated restraint [121], electric shock [122], chronic-unpredictable stress [72], and social stress [123]. These different types of stressors can vary in intensity, duration, and predictability, all of which may result in specific effects on the mother and fetus. For example, a review of animal studies [124] found that two types of prenatal stress—restraint stress and chronic variable stress—predicted generalized outcomes (e.g., increased stress-related behaviour, increased glucocorticoid stress response) and stress-type specific ones (e.g., increased CRF expression, increased *NR3C1* DNA methylation for chronic variable stress only).

Relatedly, the timing of prenatal stress also represents an important consideration. Despite significant knowledge regarding the time course of fetal brain development, there are relatively few studies that investigate the gestational timing of prenatal stress and offspring functioning. Nevertheless, there are suggestions that perturbations early in pregnancy are likely to produce more severe neurological insults than later stressors, perhaps via effects on placental functions and neural organization [125]. Work by Davis and Sandman [126] shows that higher maternal cortisol levels early in gestation predicted poorer cognitive development in offspring but better development when occurring late in gestation. Intriguingly, the opposite seems true when it comes to prenatal-stress effects on emotional and behavioural problems during childhood [127]. Other work investigating administration of antenatal corticosteroid treatment (usually between the late second trimester and early third trimester) predicted greater incidences of mental and behavioural disorders among sibling pairs [128].

Most importantly perhaps given the focus of this report, yet other evidence indicates that environmental sensitivity in response to prenatal stress may depend on the intensity of the prenatal stress. Hartman et al. [129] examined the effects of prenatal stress (i.e., maternal anxiety/ depression and stressful life events during pregnancy) on postnatal sensitivity using data from a large and representative prospective study of Norwegians. When using extreme groups to quantify prenatal stress (i.e., very high/very low), children exposed to greater prenatal stress were more sensitive, compared to children less prenatally stressed, to effects on child internalizing behaviour of mother’s postnatal maternal depressive/anxiety symptoms [129]. However, and perhaps surprisingly, in an analysis using the full sample, children exposed to the *least* prenatal stress evinced greater susceptibility to postnatal maternal depressive/anxiety symptoms on externalizing and internalizing behaviour.

Animal work also documents that the effect of prenatal stress on offspring depends on stress intensity. In one such experiment, rats were subjected to the same prenatal stress paradigm (i.e., pregnant dam placed on elevated platform) but differing intensities (more or less platform time). Intensity predicted distinctive patterns of offspring development (e.g., brain weight, sensorimotor development, DNA methylation levels) [130]. Taken together, it appears that prenatal stress may have differing effects on sensitivity depending on the intensity of prenatal stress.

## Unknowns in the prenatal programming equation

It should be clear that there is substantial evidence that environmental exposures as early as during the prenatal period appear to affect sensitivity to environmental effects. Critically, the work summarized herein for illustrative purposes suggests that individuals exposed to prenatal stress are not only more vulnerable to postnatal adversity but also respond more strongly to positive environmental quality such as high-quality maternal care, consistent with theories highlighted at the beginning of this report. Needless to say, multiple questions remain to be addressed in future research.

The reviewed work suggests that prenatal stress increases environmental sensitivity. However, most work on prenatal programming tends to examine direct effects of the prenatal environment on postnatal outcomes, not accounting for interactive effects with the postnatal environment. And even when interactive effects are examined, postnatal conditions are often limited to a negative or neutral condition instead of the full range of environmental quality from adverse to supportive (i.e., not just benign). This focus, which reflects the mainstream view, emphasizes pathological development (e.g., anxiety, depression, cognitive disorders, poor health), thereby leaving little opportunity to illuminate the postnatal conditions under which prenatal stress may promote more positive development rather than impede it. Clearly, more research is needed to investigate prenatal programming of sensitivity to both detrimental and beneficial aspects of the postnatal environment.

One important limitation of most human studies is that detected associations between prenatal exposure and child development are likely confounded by genes shared by mother and child. This is a particular concern when prenatal stress is based on maternal reports of depression and anxiety (or measures of physiological stress). Many investigations try to account for this by controlling for postnatal maternal depression. But this may not be sufficient in order to discount the alternative explanation that women who report more stress during pregnancy also tend to have more stress-reactive children due to shared genes. Indeed, a unique human cross-fostering study comparing women with their own versus those with donated eggs for in-vitro fertilization found that some prenatal effects reflect inheritance rather than programming, including effects of prenatal maternal stress on children’s postnatal anxiety [131, 132]. Future studies should further disentangle inherited and environmental effects in prenatal programming studies by considering measured genes (i.e., applying genome-wide methodologies) in both child and mother. This is especially important given that environmental sensitivity has been shown to have a substantial genetic component [6].

Some theories stipulate that exposure to different environmental quality gives rise to different types of sensitivity [2, 4, 133]. For example, being exposed to a predominately adverse environment may foster the development of a more vigilant and stress-reactive type of sensitivity, whereas growing up in a consistently supportive context may result in a vantage sensitivity type that tends to benefit disproportionately from positive aspects of the environment [4]. Although first evidence supports aspects of this hypothesis [134], further work is needed to explore the existence of different sensitivity types and investigate whether different qualities of the prenatal environment play a role in shaping different types of sensitivity.

Another issue concerns the stability of sensitivity over time, that is, whether prenatally programmed environmental sensitivity is stable across life or whether it can change in response to the postnatal context. The stability of sensitivity across development has not been investigated in depth yet. Intriguingly, first evidence suggests that observer-rated sensitivity in three year old children can change within a year as a function of environmental quality [135], a result not inconsistent with *Biological Sensitivity to Context* thinking. Future studies should investigate to what degree prenatally programmed sensitivity is stable across subsequent developmental periods and identify the postnatal exposures that are likely to reverse or modify prenatally programmed environmental sensitivity.

Another important area for future research is to determine what specific prenatal conditions lead to increased environmental sensitivity. As mentioned previously, there is evidence to suggest that type, timing, and intensity all matter for prenatal stress effects. However, most of these studies are limited and have not been designed to test prenatal programming of environmental sensitivity. For example, few inquiries have compared prenatal factors such as varying levels of intensity or different types of prenatal stress and their relative influences on development. Furthermore, there is no systematic review, to our knowledge, of specific features of prenatal stress and their association with increased environmental sensitivity. It would be an important endeavour to systematically catalogue these differences indicating the type, timing, and intensity of prenatal stressors, postnatal environmental measures, associated outcomes, and effect sizes in relation to environment sensitivity. In this way, researchers may determine whether effects of prenatal stress on environmental sensitivity are more generalized or specific. It might be the case that extreme levels of prenatal stress confer only negative effects on the fetus or that non-linear relations exist similar to Selye’s stress theory [136].

## Implications

Besides providing a broader conceptualization regarding the developmental impact of prenatal exposures compared to the traditional perspective [40], the current review specifically suggests that prenatal adversity does not necessarily result in pathology but can actually contribute to positive development when paired with a nurturing postnatal context. This implies that early support for the child and his/her family in the postnatal period could be especially beneficial and effective for children with a history of prenatal distress. At the same time, these children are at heightened risk for maladaptive development if growing up under adverse postnatal conditions. Hence, early intervention (e.g., programmes promoting supportive parenting) for prenatally stressed children is of great importance because it may not only prevent development of problematic outcomes but could facilitate and promote positive development.

In conclusion and in contrast to the traditional pathology-based perspective on prenatal programming effects, exposure to prenatal stress can increase general environmental sensitivity to the quality of the postnatal environment. According to a growing literature focused on animals and humans, individuals with a history of prenatal distress have not only been found to be more vulnerable to the negative effects of postnatal adversity but also disproportionately sensitive to the developmental benefits of a supportive postnatal context.
